# Distinct T and NK cell populations may serve as immune correlates of protection against symptomatic pandemic influenza A(H1N1) virus infection during pregnancy

**DOI:** 10.1371/journal.pone.0188055

**Published:** 2017-11-16

**Authors:** Miloje Savic, Jennifer L. Dembinski, Ida Laake, Olav Hungnes, Rebecca Cox, Fredrik Oftung, Lill Trogstad, Siri Mjaaland

**Affiliations:** 1 Infection Control and Environmental Health, Norwegian Institute of Public Health, Oslo, Norway; 2 KG Jebsen Centre for Influenza Vaccine Research, Oslo-Bergen, Norway; 3 Influenza Centre, Department of Clinical Science, University of Bergen, Bergen, Norway; 4 Department of Research and Development, Haukeland University Hospital, Bergen, Norway; The University of Chicago, UNITED STATES

## Abstract

Maternal influenza infection during pregnancy is associated with increased risk of morbidity and mortality. However, the link between the anti-influenza immune responses and health-related risks during infection is not well understood. We have analyzed memory T and NK cell mediated immunity (CMI) responses in pandemic influenza A(H1N1)pdm09 (pdm09) virus infected non-vaccinated pregnant women participating in the Norwegian Influenza Pregnancy Cohort (NorFlu). The cohort includes information on immunization, self-reported health and disease status, and biological samples (plasma and PBMC). Infected cases (N = 75) were defined by having a serum hemagglutination inhibition (HI) titer > = 20 to influenza pdm09 virus at the time of delivery, while controls (N = 75) were randomly selected among non-infected pregnant women (HI titer <10). In ELISpot assays cases had higher frequencies of IFNγ^+^ CD8^+^ T cells responding to pdm09 virus or conserved CD8 T cell-restricted influenza A virus epitopes, compared to controls. Within this T cell population, frequencies of CD95^+^ late effector (CD45RA^+^CCR7^-^) and naive (CD45RA^+^CCR7^+^) CD8^+^ memory T cells correlated inversely with self-reported influenza illness (ILI) symptoms. ILI symptoms in infected women were also associated with lower numbers of poly-functional (IFNγ^+^TNFα^+^, IL2^+^IFNγ^+^, IL2^+^IFNγ^+^TNFα^+^) CD4^+^ T cells and increased frequencies of IFNγ^+^CD3^-^CD7^+^ NK cells compared to asymptomatic cases, or controls, after stimulation with the pdm09 virus. Taken together, virus specific and functionally distinct T and NK cell populations may serve as cellular immune correlates of clinical outcomes of pandemic influenza disease in pregnant women. Our results may provide information important for future universal influenza vaccine design.

## Introduction

During the 2009 influenza pandemic, pregnant women had an increased risk of severe influenza illness and adverse pregnancy outcomes [1–3], and 12% of pregnancy-related deaths were attributed to pandemic infection [4].

The mechanisms behind influenza infection-related mortality and morbidity in pregnancy are still poorly understood, however it is speculated that immune modulation required for fetal tolerance may be one of the major contributing factors [5–7]. Murine models suggest that increased mortality and morbidity may be due to either a deficiency in cellular immunity or to a hyper-reactive immune response to inflammatory cytokines and chemokines that govern cellular infiltration and exacerbation of symptoms [8, 9]. In humans, the frequencies of monocytes and dendritic cells were increased whereas natural killer (NK) and T cells frequencies were decreased [10]. NK and T cells from pregnant women had reduced production of interferon (IFN)-γ and macrophage inflammatory protein (MIP)-1β [10, 11]. Recently, CD8^+^ effector cells and T regulatory cells (Tregs) at the fetal-maternal interface were implicated as modulators of fetal-immune tolerance and antiviral immunity [12–14].

Due to repeated exposure to circulating influenza strains in adults cellular immunity is dominated by memory T cell responses. In the case of the 2009 pandemic, the new influenza H1N1 strain was a result of reassortment between swine, human and avian influenza A strains [15]. This strain contained several novel epitopes able to elicit naive CD4^+^ and CD8^+^ T cell responses with potentially different dynamics to those existing from pre-established (heterosubtypic) memory responses against the conserved epitopes. Heterosubtypic immunity is mainly mediated by cross-reactive cytotoxic CD8^+^ T cells [16–18], and pre-existing influenza-specific CD4^+^ and CD8^+^ T cells effectively cross-reacted to the newly emerged influenza A(H1N1)pdm09 virus [19–23].

We analyzed memory cellular immune responses in relation to influenza-like illness (ILI) symptoms after pandemic influenza A(H1N1)pdm09 virus infection during pregnancy. Our results showed that virus specific and functionally distinct T and NK cell populations may serve as cellular memory-immune correlates of favorable clinical outcomes of pandemic influenza disease.

## Materials and methods

### Study participants and design

The Norwegian Influenza Pregnancy Cohort (NorFlu) Study was established during the influenza A(H1N1)pdm09 pandemic in Norway. Enrollment of pregnant women to NorFlu study started in February 2010. Women with their last menstrual period between 1^st^ June 2009 and 31^st^ December 2009, and therefore pregnant during the H1N1 pandemic, were invited to participate at the time of the routine ultrasound examination around gestational week 17–20. The participation rate was 41%, and all participants signed a written consent.

The project protocol was reviewed and approved by the institutional review board at the NIPH and Regional Committee for Medical and Health Research Ethics South East, Norway (study reference numbers 2009/2165 and 2010/2937). All participants have signed a written consent.

A nested case-control study of non-vaccinated pregnant women included infected cases and non-infected controls during the pandemic peak in Norway (1.10.2009.– 31.12.2009.) (S1 Table). Cases (N = 75) were defined by having a serum hemagglutination inhibition (HI) titer > = 20 to A(H1N1)pdm09 virus at the time of delivery. Controls (N = 75) were randomly selected among non-infected pregnant women with an HI titer <10 at delivery. Pre-existing protective HI titers to the influenza A(H1N1)pdm09 virus were low in the Norwegian population prior to the pandemic, including women of fertile age [24].

### Outcomes and symptom score

Cases were further categorized based on self-reported questionnaires as 1) symptomatic–reporting influenza-like illness (ILI) symptoms during the pandemic peak (October–December 2009): fever and cough or fever and sore throat, or 2) asymptomatic–reporting no ILI symptoms. Further, a symptom score was designed by totaling the weight for each of the canonical ILI symptoms: fever, cough, sore throat, rhinitis, headache, and myalgia, with a weight of 1, 4 or 6, if the reported duration of symptoms was 0–2, 3–5 or more than 5 days, respectively. Self-report of many symptoms, more severe illness and longer duration of illness were associated with higher HI titers (p<0.05 for all comparisons, manuscript in preparation).

### Pandemic virus and epitope libraries

In all ELISpot and flow cytometry assays a H_2_O_2_ inactivated A/California/07/2009(H1N1)pdm09 (pdm09) virus was used at 75 hemagglutination units (HAU) for stimulation of PBMC [25]. In addition, a total of seven influenza A epitope libraries were used as previously described [26]. In brief, conserved influenza A epitopes were divided in four universal influenza A peptide libraries according to MHC class I and II restriction (uCD8 and uCD4, respectively). The libraries were divided according to epitope origin: 1) external viral proteins–uCD8e and uCD4e, and 2) internal viral proteins–uCD8i and uCD4i. Peptides unique for the pandemic A(H1N1)pdm09 virus were similarly grouped in three libraries: pCD4e, pCD8i and pCD4i (pCD8e library did not include unique peptides; more details in [26]).

### *Ex vivo* enzyme-linked IFNγ/Granzyme B immunospot assay (ELISpot)

ELISpot assays were performed as previously described [26], with additional information provided in Supplementary Materials and Methods (S1 and S2 Figs, S1 and S2 Files).

### Flow cytometry assays

Flow cytometry assays, and gating strategies, were performed as previously described [26–28], with additional information provided in Supplementary Materials and Methods (S7 and S8 Figs, S2 Table, S1 and S2 Files). Cells were analyzed on a BD LSRII flow cytometer using FACS Diva and FlowJo (TreeStar) software. Cells were gated according to forward- and side-scatter plots, and 40,000 CD4^+^ T cells, or 25,000 CD3^-^ cells, were acquired. All functional values are presented as a fold change over a negative control, assuming synergistic rather than an additive effect [29].

### Statistical analyses

The frequencies of antigen-specific T cells in ELISpot assays between exposure groups were compared using Wilcoxon rank-sum test. The Kruskal-Wallis test followed by Dunn’s ‘post-hoc’ test was used to analyze differences in antigen-specific T cells frequency fold change among the three exposure groups in flow cytometry experiments.

Presence of individual ILI symptom and the frequency of IFNγ and Granzyme B producing PBMC, and frequency of T and NK cells analyzed by flow cytometry were correlated using point biserial correlation (i.e. to correlate a dichotomous and a continuous variable). Total symptom score and cell frequencies were analyzed by Spearman rank correlation (i.e. to correlate two continuous variables).

## Results

### Frequency of IFNγ-secreting CD8^+^ T cells was correlated with asymptomatic infection

**Fig 1:**
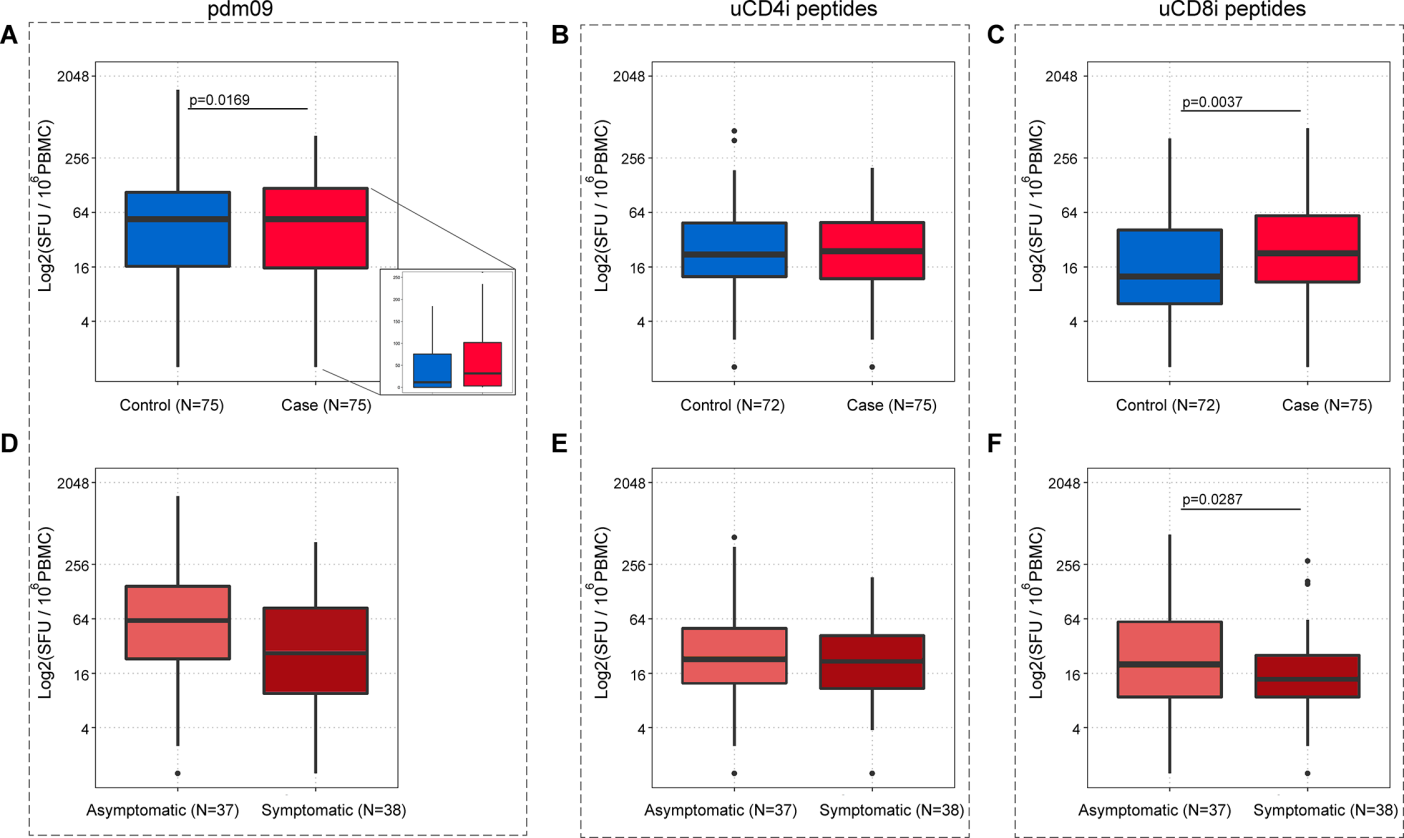
Frequency of IFNγ secreting PBMC measured in ELISpot assays after stimulation with inactivated pdm09 virus. (A, D), uCD4i, (B, E), and uCD8i (C, F) epitope library. Top row represents differences among cases and controls (A, B, C), bottom row represents differences between asymptomatic and symptomatic cases (D, E, F). Inlet in figure A zooms on the range of cell frequencies presented on a linear scale (p values, Wilcoxon-Mann-Whitney test).

Cases had a higher median frequency of IFNγ-secreting cells compared to controls after stimulation with the whole inactivated pdm09 virus (p = 0.0169), or the uCD8i epitope library (p = 0.0037), but not after stimulation with the uCD4i epitope library (Fig 1A, 1B and 1C). Further analysis of cases, based on reported illness symptoms, showed a higher frequency of IFNγ-secreting cells in cases reporting no symptoms (N = 37) compared to those who experienced ILI symptoms (N = 38) (p = 0.0287), after stimulation with the uCD8i epitope library (Fig 1F). The frequency of this cell population was inversely correlated with the ILI symptoms (rho = -0.2303, p = 0.0468), suggesting a protective role of these cells against the symptomatic pdm09 infection. The levels of anti-pdm09 virus antibodies were comparable between symptomatic and asymptomatic cases (p = 0.5180, data not shown).

In contrast, in infected symptomatic women (N = 38), the total symptom score during ILI had modest positive correlation with the frequency of IFNγ secreting cells after stimulation with universal internal CD4 epitopes (uCD4i, rho = 0.3173, p = 0.0491) (Fig 1E). Collectively, our results suggest somewhat opposing roles of IFNγ-secreting T cells: protective effect against the symptomatic illness mediated by cells responding to the universal CD8 internal influenza A epitopes, while there was a potentially symptoms induction by cells stimulated by the universal CD4 internal viral epitopes.

### Different populations of memory T cells were inversely correlated with ILI symptoms

After stimulation with the pdm09 virus we observed increased frequency fold change of CD95^+^ CD4^+^ and CD8^+^ naive T cells among asymptomatic cases compared to those reporting symptoms (S3A and S3B Fig). The frequency of CD95^+^CD4^+^ naive T cells was inversely correlated with most self-reported ILI symptoms: fever (rho = -0.4794, p = 0.0603), cough (rho = -0.3795, p = 0.1471), and sore throat (rho = -0.5207, p = 0.0380). Similarly, negative correlations were observed between the frequency of CD95^+^CD8^+^ naive T cells and self-reported ILI symptoms: fever (rho = -0.4320, p = 0.0947), cough (rho = -0.3544, p = 0.1781), and sore throat (rho = -0.5115, p = 0.0428). In addition, the frequency of CD95^+^CD8^+^ naive T cells was negatively correlated with self-reported fever (rho = -0.4844, p = 0.0572), after stimulation with conserved influenza A peptides (uCD8i) (S4 Fig).

**Fig 2:**
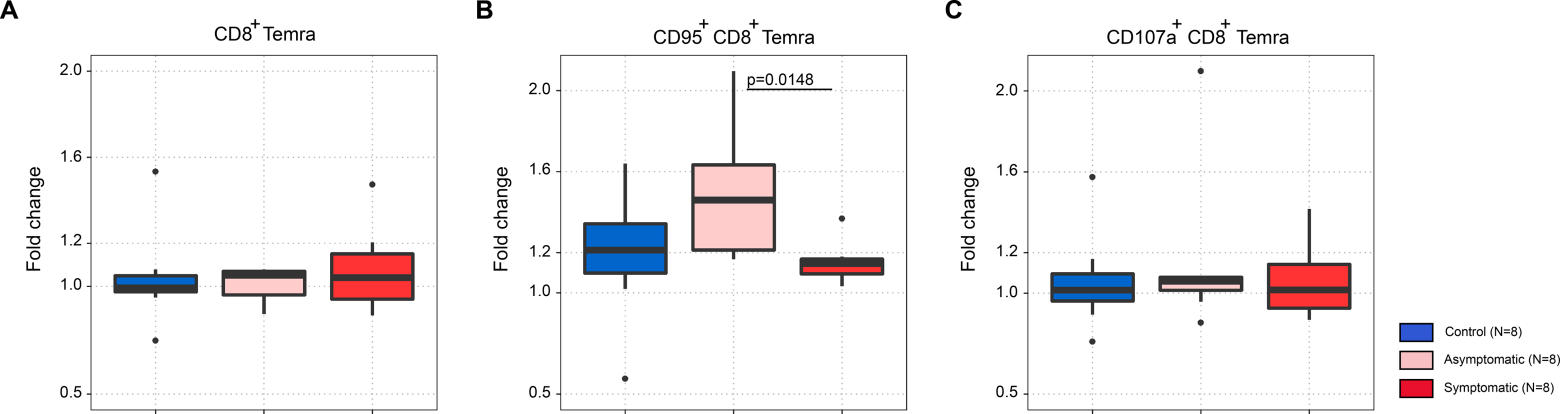
CD95^+^ CD8^+^ Temra cells as cellular immune correlates of protection against symptomatic ILI after exposure to pdm09 virus in pregnancy. PBMC samples were stimulated ex vivo, y-axis in all panels shows cell frequency fold change over unstimulated control: (A) CD8^+^ Temra, (B) CD95^+^ CD8^+^ Temra, (C) CD107^+^ CD8^+^ Temra (p vlaues, Dunn’s Kruskal-Wallis post hoc test).

Symptomatic cases had a reduced frequency of CD95^+^CD8^+^ central memory T cells (Tcm) compared to asymptomatic cases (p = 0.0929). Late effector memory CD8^+^ T cells (CD8^+^ Temra) that express CD95 marker were significantly reduced in symptomatic cases compared to asymptomatic cases (p = 0.0148), after stimulation with the pdm09 virus (Fig 2B), which was not observed for CD8^+^ nor CD107a^+^CD8^+^ Temra cells (Fig 2A and 2C). The frequency of CD95^+^CD8^+^ Temra, but not CD107a^+^CD8^+^ Temra cells, was inversely correlated with self-reported fever (r = -0.5794, p = 0.0187), headache (r = -0.4951, p = 0.0798), cough (r = -0.4951, p = 0.0512), and sore throat (r = -0.606, p = 0.0128), indicating a protective effect against symptomatic ILI during pregnancy. Furthermore, after stimulation with the uCD8i epitope library, the frequency of CD95^+^CD8^+^ Temra cells was inversely correlated with fever (r = -0.5126, p = 0.0423), suggesting cell-mediated cross-protection against the symptomatic pandemic influenza A(H1N1)pdm09 virus infection.

### Multifunctional CD4^+^ and CD8^+^ T cells were associated with favorable ILI symptoms

**Fig 3:**
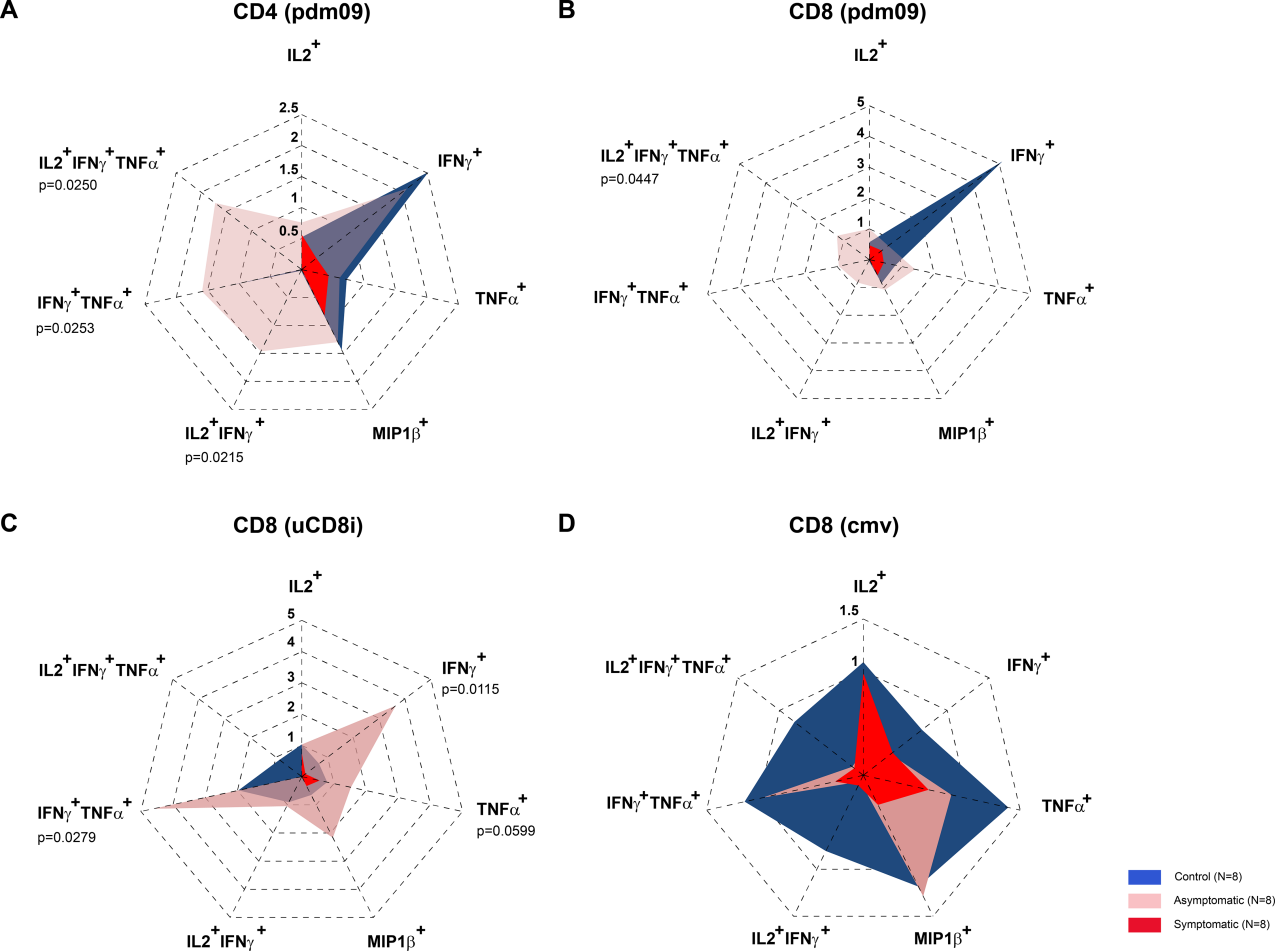
Effector functions of CD4^+^ and CD8^+^ T cells. Median cell frequency fold changes of (A) CD4^+^, (B) CD8^+^ T cells after stimulation with the pdm09 virus, and median CD8^+^ T cell frequency fold change after stimulation with (C) uCD8i, (D) CMV epitopes. Dotted lines in graphs represent the fold change level (p values, Dunn’s Kruskal-Wallis post hoc test).

We analyzed the effector functions of the CD4^+^ and CD8^+^ T cell responses after stimulation with the pdm09 virus or uCD8i peptides. We observed increased frequency of IFNγ^+^ and MIP1β^+^ CD4^+^ T cells in controls and asymptomatic cases compared to symptomatic cases after stimulation with the pdm09 virus (2.5 and 1.5 fold change increase, respectively) (Fig 3A). In addition, median fold changes in frequency of IL2^+^CD4^+^ and TNFα^+^CD4^+^ T cells were lower in symptomatic cases compared to asymptomatic cases (Fig 3A). The increase in frequency fold change of IFNγ^+^TNFα^+^CD4^+^ T cells was significantly higher in asymptomatic cases compared to symptomatic cases (p = 0.0253), after stimulation with the pdm09 virus (Fig 3A), which was also observed in the IL2^+^IFNγ^+^ and IL2^+^IFNγ^+^TNFα^+^ CD4^+^ T cell compartments (p = 0.0215 and p = 0.0250, respectively).

In the CD8^+^ T cell compartment single cytokine producing cells: IFNγ^+^, IL2^+^, TNFα^+^, or MIP1β^+^ had lower frequency fold changes in symptomatic cases compared to asymptomatic cases after stimulation with the pdm09 virus (Fig 3B). The frequency of triple cytokine producing IL2^+^IFNγ^+^TNFα^+^ CD8^+^ T cells was significantly higher in asymptomatic compared to symptomatic cases (p = 0.0447). Frequency changes of single cytokine producing (IFNγ^+^, MIP1β^+^, TNFα^+^), and double cytokine producing (IFNγ^+^TNFα^+^) CD8^+^ T cells specific for uCD8i epitopes were higher in asymptomatic cases compared to symptomatic cases (~3.5, 2, ~2, and >4 fold, respectively) (Fig 3C). Stimulation with HLA class I restricted CMV peptides did not induce significant changes in comparable CD8^+^ T cell populations (Fig 3D), further strengthening the observation that the effector CD8^+^ T cell responses were influenza A specific with a potential to provide heterosubtypic immunity since the uCD8i peptide epitope library represents a collection of conserved epitopes present in influenza A strains spanning the period from 1934 to 2009 [26].

### Different NK cell populations were associated with ILI symptoms

**Fig 4:**
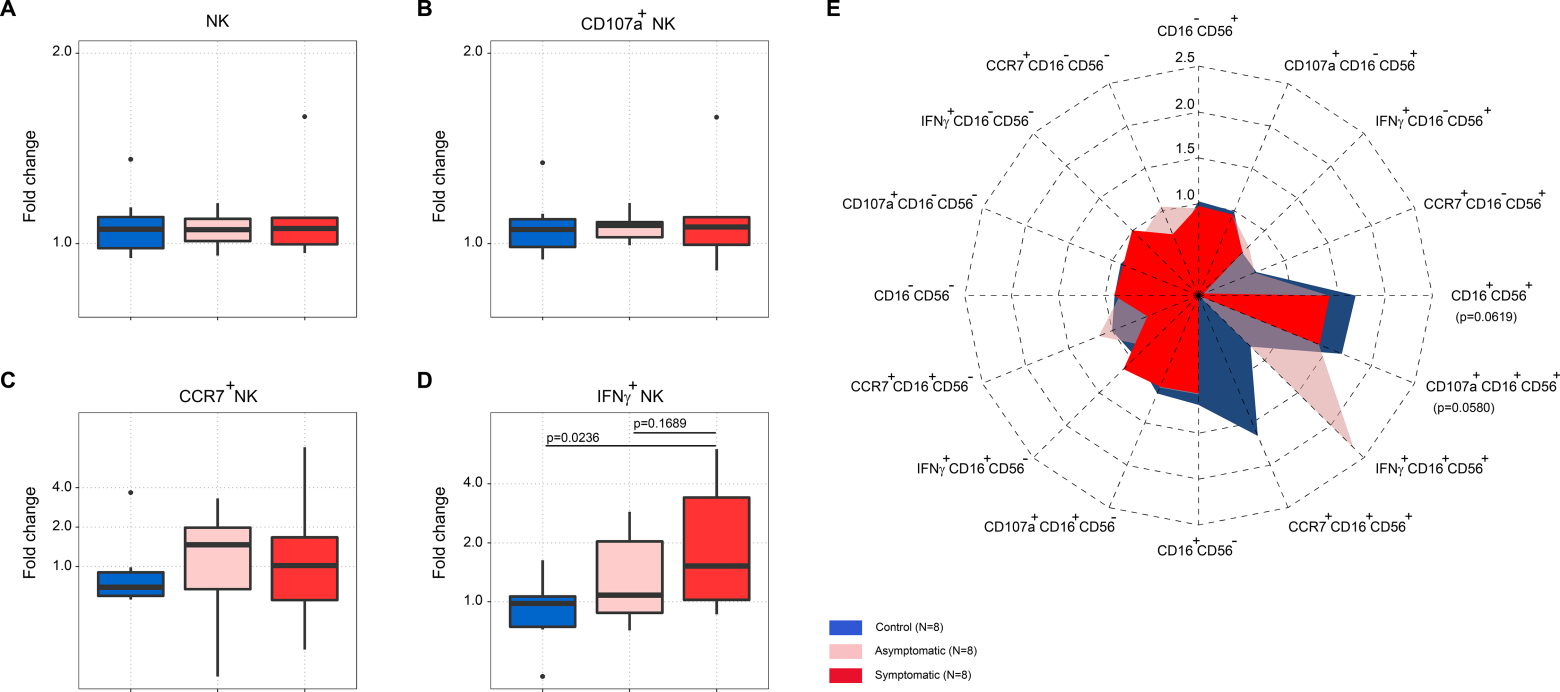
Cell frequency fold change among controls, asymptomatic and symptomatic cases after the pdm09 virus stimulation. (A) total NK cells, (B) CD107a^+^ NK cells, (C) CCR7^+^ NK cells, (D) IFNγ^+^ NK cells, (E) radar graph of specific NK cell populations characterized by the surface markers CD16 and CD56, and positive for CD107a, CCR7 or IFNγ expression. Dotted lines in graph E represent the fold change level (p values, Dunn’s Kruskal-Wallis post hoc test in D, factorial ANOVA in E).

The cell frequencies of the total NK and CD107a^+^ NK cells were comparable among three groups of women (Fig 4A and 4B). Cases had higher frequencies of CCR7^+^ and IFNγ^+^ NK cells, with symptomatic cases having increased frequency of IFNγ^+^ NK cells compared to asymptomatic cases (p = 0.1689), or controls (p = 0.0236) after stimulation with the pdm09 virus (Fig 4C and 4D).

The control group had the highest cell frequency fold change of CD16^+^CD56^+^ NK cells after stimulation with the whole inactivated pdm09 virus compared to symptomatic cases (p = 0.0619), which was also observed in CCR7^+^CD16^+^CD56^+^ and CD107a^+^CD16^+^CD56^+^ (p = 0.0580) NK cell compartments (Fig 4E). An almost 2.5 fold increase in median cell frequency of IFNγ^+^CD16^+^CD56^+^ NK cells was observed in half of the asymptomatic cases compared to controls, while this cell population was almost completely absent in symptomatic cases. In contrast, symptomatic cases had a slightly increased median frequency of IFNγ^+^CD16^+^CD56^-^ NK cells, compared to asymptomatic cases or controls.

Finally, we analyzed the possible correlations in cell frequencies among effector CD4^+^ and CD8^+^ T cells and different populations of NK cells (S5 Fig). In the control group, prominent negative correlations within the NK cell compartment were observed: CD16^+^CD56^-^ NK cells were negatively correlated with CD16^-^CD56^-^ and CD107a^+^CD16^-^CD56^-^ NK cells. We also found negative correlations between CD16^+^CD56^-^ NK and IL2^+^CD4^+^ T cells. Among asymptomatic cases, more positive correlations were observed between CD4^+^ and CD8^+^ T cell compartments, and increasing number of negative correlations between cells in the CD16^-^CD56^-^ NK and CD8^+^ T cell compartments. In contrast, symptomatic cases showed multiple positive correlations between cells in the IFNγ^+^ and CD107^+^CD16^-^CD56^-^ NK and IFNγ^+^TNFα^+^CD4^+^ T cell compartments. Also, cell frequencies of the CD107a^+^CD16^+^CD56^+^ and CD107a^+^CD16^+^CD56^-^ NK and TNFα^+^, IFNγ^+^ and TNFα^+^IFNγ^+^ CD4^+^ T cell compartments were positively correlated, suggesting a possible mechanism of reinforcing the inflammatory loop between innate and adaptive CMI responses. The overall differences in cellular correlations of the CMI response networks between the three groups of pregnant women were statistically significant (p<0.0001, S5 Fig), indicating an association between altered CMI responses and disease severity in pregnancy.

## Discussion

We analyzed the relationship between T and NK cell-mediated immunity and disease severity in pregnant women infected with influenza A(H1N1)pdm09 virus during the 2009 pandemic in Norway. Influenza infected women without ILI symptoms had a higher magnitude of IFNγ producing CD8^+^ T cells, compared to those reporting symptoms, as measured in ELISpot assays after stimulation with the pdm09 virus or peptides representing conserved CD8^+^ T cell epitopes (uCD8i). These antigen-specific CD8^+^ T cells are of particular interest due to their ability to provide cross-protective immunity against a range of influenza viruses [20, 21, 30, 31], and may provide the basis for universal influenza vaccine development [16, 32]. The inverse correlation observed between reported symptoms and frequency of IFNγ-secreting T cells indicates a putative protective effect of this cell population against symptomatic pandemic influenza illness during pregnancy. A similar observation has been reported previously: higher frequencies of pre-existing T cells specific for influenza A CD8 epitopes were found in individuals who reported mild ILI symptoms [21]. Nevertheless, it must be noted that the samples in our study were collected at the time of delivery, which means that the average time interval between exposure to pandemic influenza A(H1N1)pdm09 virus and the collection of the blood samples was 7.5 months. However, due to the fact that the average human is exposed on multiple occasions to influenza infection during their lifetime, and that our universal CD8^+^ T cell epitope library contains the most conserved influenza A epitopes, it is reasonable to assume that some, if not most, of the IFNγ^+^ CD8^+^ T cells, responding to such epitopes, were present before the pandemic and thus may have provided cross-protective immunity against the 2009 pandemic strain.

We further examined the association between different memory T cell subtypes and disease severity after exposure to the A(H1N1)pdm09 virus. Our results suggest that the CD95^+^CD8^+^ late effector memory T cells (Temra) may serve as a potential memory-immune correlate of protection against symptomatic pandemic influenza A illness in pregnant women. The finding that CD95^+^ Temra cells after stimulation with both pdm09 virus and the uCD8i epitope library were inversely correlated with fever, suggests a cross-protective role for this cell population against the pandemic virus as well as a range of other influenza A strains. Previous reports have identified various pre-existing CD4^+^ and CD8^+^ T cell populations as cellular immune correlates of protection against symptomatic influenza disease. Wilkinson and coworkers, identified IFNγ^+^CD4^+^ memory T cells (CD27^+^CD45RO^-^CD4^+^) with cytotoxic potential against conserved CD4 epitopes in a human challenge study [23]. In contrast, Sridhar et al. identified pre-existing IFNγ^+^CD8^+^ Temra cells, specific for conserved influenza A viral epitopes, as cellular immune correlates of protection against community acquired symptomatic pandemic influenza [21].

Abundant expression of the CD95 marker on naive memory T cells was recently described on so-called memory stem T cells (T_SCM_), which were characterized by enhanced self-renewal and multipotency, increased proliferative capabilities and rapid acquisition of effector functions after antigen stimulation [33]. We observed increased frequencies of CD95^+^CD4^+^ and CD95^+^CD8^+^ naive memory T cells in asymptomatic compared to symptomatic cases. Both CD95^+^ naive memory CD4^+^ and CD8^+^ T cells, which are phenotypically similar to T_SCM_, were inversely correlated with occurrence of ILI symptoms in A(H1N1)pdm09 virus exposed pregnant women. Therefore it is warranted to further investigate T_SCM_ cells role in CMI against influenza and implications for the design of T cell-based influenza vaccine.

Both the magnitude and quality of antiviral CD4^+^ and CD8^+^ T cell responses are critical for the control of viral infection and the course of (symptomatic) illness. We observed effector responses in the CD4^+^ T cell compartment after stimulation with the A(H1N1)pdm09 virus, which was in accordance with a previous report showing that pregnant women can elicit robust T cell responses against influenza A viruses [34]. Changes in the frequency of multifunctional IL2^+^IFNγ^+^, IFNγ^+^TNFα^+^ and IL2^+^IFNγ^+^TNFα^+^ CD4^+^ T cells were higher in asymptomatic cases as compared to both symptomatic cases and controls. Multiple-cytokine producing CD4^+^ T cells were previously described as highly potent cytokine producers that could provide better co-stimulatory functions to CD8^+^ T cells and B cells: characteristics that enable superior antiviral responses [35]. Our results reaffirm the notion that the characteristics of multi-cytokine producing CD4^+^ T cells constitute an important aspect of the complex cellular immunity network that provides cues for illness severity during pregnancy.

Effector functions in the CD8^+^ T cell compartment were characterized by reduced changes in frequency of IFNγ^+^CD8^+^ T cells in cases compared to controls, and generally lowered frequencies of CD8^+^ T cells among symptomatic compared to asymptomatic cases. Multifunctional CD8^+^ T cells were more prominent in asymptomatic cases compared to controls or symptomatic cases. We observed more than a 4 fold change in IFNγ^+^TNFα^+^ CD8^+^ T cell frequencies after stimulation with conserved CD8 epitopes in asymptomatic compared to symptomatic cases, which was also true for IFNγ^+^ and TNFα^+^ CD8^+^ T cell populations. These CD8^+^ T cell populations, specific for the conserved CD8 epitope library, could provide heterosubtypic cellular immunity against different influenza A infections [36]. In general, asymptomatic cases had more robust multifunctional CD4^+^, and to some extent CD8^+^ T cell responses, as compared to their symptomatic counterparts, which could partly explain the difference in the symptomatic course of illness, and points towards the growing evidence of crucial role(s) of CD4^+^ T cells in anti-influenza response [23, 37].

Finally, we analyzed the effector functions of circulating NK cells. Cases had higher changes in frequency of CCR7^+^ and IFNγ^+^ NK cells compared to controls. A notable feature was a steady increase in cell frequency in the IFNγ^+^ NK cell compartment when moving from controls to asymptomatic to symptomatic cases after stimulation with the pdm09 virus. Our results are in accordance with previous report showing increased IFNγ^+^ NK cell frequencies in pregnant women following influenza infection or vaccination [34]. We further dissected the NK cell compartment according to expression of CD16 and CD56 markers. Compared to controls, cases had no CCR7^+^CD16^+^CD56^+^ NK cells. We observed more than 2 fold increase of IFNγ^+^CD16^+^CD56^+^ NK cell frequency in asymptomatic cases compared to controls, albeit only in half of the analyzed asymptomatic cases, while this NK cell population was completely absent in symptomatic cases. Double positive NK cells are characterized by a permanent state of readiness for immediate response, and they are implicated in antibody-dependent cellular cytotoxicity (ADCC) and can rapidly produce IFNγ upon activation [38]. All these features implicate IFNγ^+^CD16^+^CD56^+^ NK cells as part of a cellular network of immune correlates of protection.

Strengths of our study are reflected in the uniqueness of the biobank that contains plasma, PBMC, DNA and RNA from women pregnant during the pandemic peak in Norway. Also, extensive data collection about the immunization, health and disease status through national health registries and questionnaires allows analyzing associations between self-reported ILI symptoms and CMI. However, limitations are reflected in an inability to establish baseline responses due to unpredictable occurrence of the pandemic influenza and the timing of pregnancy and sample collection; furthermore the study had limited samples available. Samples were from the peripheral blood, thus the overall picture of cellular immune effector functions and memory does not reflect tissue-specific immunity (e.g. upper airways or lungs). Finally, most women were exposed during the first trimester of pregnancy whereas blood samples were collected at birth. The observed cell frequencies of antigen specific CD4 and CD8 T cells and NK cells could have been affected by the hormonal status at labor, as it was noted previously that a rise in progesterone and estradiol levels could negatively influence cell frequencies of adaptive immunity [5, 6]. Therefore it is of great importance to continue efforts to analyze CMI in different trimesters of pregnancy specifically in the acute phase of influenza A infection.

Influenza infection in pregnancy is a complex, challenging and clinically important problem. In a community acquired infection setting, we observed qualitatively and quantitatively different CMI responses against the pandemic A(H1N1)pdm09 virus in symptomatic and asymptomatic pregnant women, which are probably major determinants of illness progression and severity. Furthermore, we have identified several antigen-specific T cell populations which potentially can be used as cellular immune correlates of cross-protection against different influenza A viruses during pregnancy. Understanding interactions between various immune cell types (e.g. T and NK cells) of the maternal immune system and the virus, and influence of immune and hormonal signals from maternal-fetal barrier, both systemically and locally, may lead to improved disease management and prevention strategies. Furthermore, our data expand the growing immunological evidence-based impetus for the design of universal influenza vaccines to induce cross-reactive T cells that could confer protection against emerging influenza viruses.

## Supporting information

S1 Fig Frequency of IFNɣ secreting PBMC among cases and controls. PBMC were stimulated with various influenza A antigen libraries: (A) pCD4i, (B) pCD4e, (C) pCD8i, (D) uCD4e, (E) uCD8e (p values, Wilcoxon-Mann-Whitney test).
(TIF)

S2 Fig Frequency of IFNγ secreting PBMC among asymptomatic and symptomatic cases. PBMC were stimulated with various influenza A antigen libraries: (A) pCD4i, (B) pCD4e, (C) pCD8i, (D) uCD4e, (E) uCD8e (p values, Wilcoxon-Mann-Whitney test).
(TIF)

S3 Fig Phenotypes of memory T cells after stimulation with pdm09 virus. Radar graphs represent median cell frequency fold change of (A) CD4^+^, (B) CD8^+^ memory T cells subsets. Dotted lines in graphs represent the fold change level (p values, Kruskal-Wallis test).
(TIF)

S4 Fig Phenotypes of CD8^+^ memory T cells after stimulation with peptide epitopes. Radar graphs represent median cell frequency fold change after stimulation with (A) uCD8i, (B) CMV epitopes. Dotted lines in graphs represent the fold change level (p values, Kruskal-Wallis test).
(TIF)

S5 Fig Circos diagrams of correlations among effector CD4^+^ and CD8^+^ T cells, and NK cells after stimulation with pdm09 virus. (A) controls, (B) symptomatic cases, (C) asymptomatic cases. Top left diagram represents visual legend for the segments orientation in each diagram–one segment corresponds to a particular immune cell subset and antigen stimulation combination, whereas left and right semicircles designate T and NK cell compartments, respectively. For clarity correlation coefficient values between -0.5 and +0.5 were not presented, values between 0.5 and 0.7 in light color, and values between 0.7 and 1 in dark color (see the bar at the top left panel) (p values, Steiger’s test).
(TIF)

S6 Fig Circos diagrams of correlation of IFNγ and Granzyme B PBMC compartments after stimulation with pdm09 and various influenza A antigens. (A) controls, (B) symptomatic cases, (C) asymptomatic cases. Top left diagram represents visual legend for the segments orientation at each diagram–one segment corresponds to a particular PBMC and antigen stimulation combination, whereas left and right semicircles designate Granzyme B and IFNγ PBMC compartments, respectively. For clarity correlation coefficient values between -0.25 and +0.25 were not presented, values between 0.25 and 0.5 in light color, and values between 0.5 and 1 in dark color (see the bar at the top left panel) (p values, Steiger’s test).
(TIF)

S7 Fig Gating strategy to define memory T-cell populations using CD45RA and CCR7 markers. SSC-A–side scatter area, FSC-A–forward scatter area, Live/Dead–aqua live/dead viability dye.
(TIF)

S8 Fig Gating strategy to define NK-cell populations using CD7, CD16 and CD56 markers. SSC-A–side scatter area, FSC-A–forward scatter area, Live/Dead–aqua live/dead viability dye.
(TIF)

S1 Table Demographic and other characteristics of cases and controls. (DOCX)

S2 Table Antibodies used for phenotyping T and NK cells. (DOCX)

S1 File Supporting information. Additional details regarding Materials and Methods, and Results section.
(DOCX)

S2 File Supporting information ELISpot and flow cytometry data. (DTA)
