# Supplementary figures and images for: Distinct T and NK cell populations may serve as immune correlates of protection against symptomatic pandemic influenza A(H1N1) virus infection during pregnancy
Source: PLoS One. 2017 Nov 16;12(11):e0188055. doi: 10.1371/journal.pone.0188055 (PMC5690673; doi:10.1371/journal.pone.0188055)

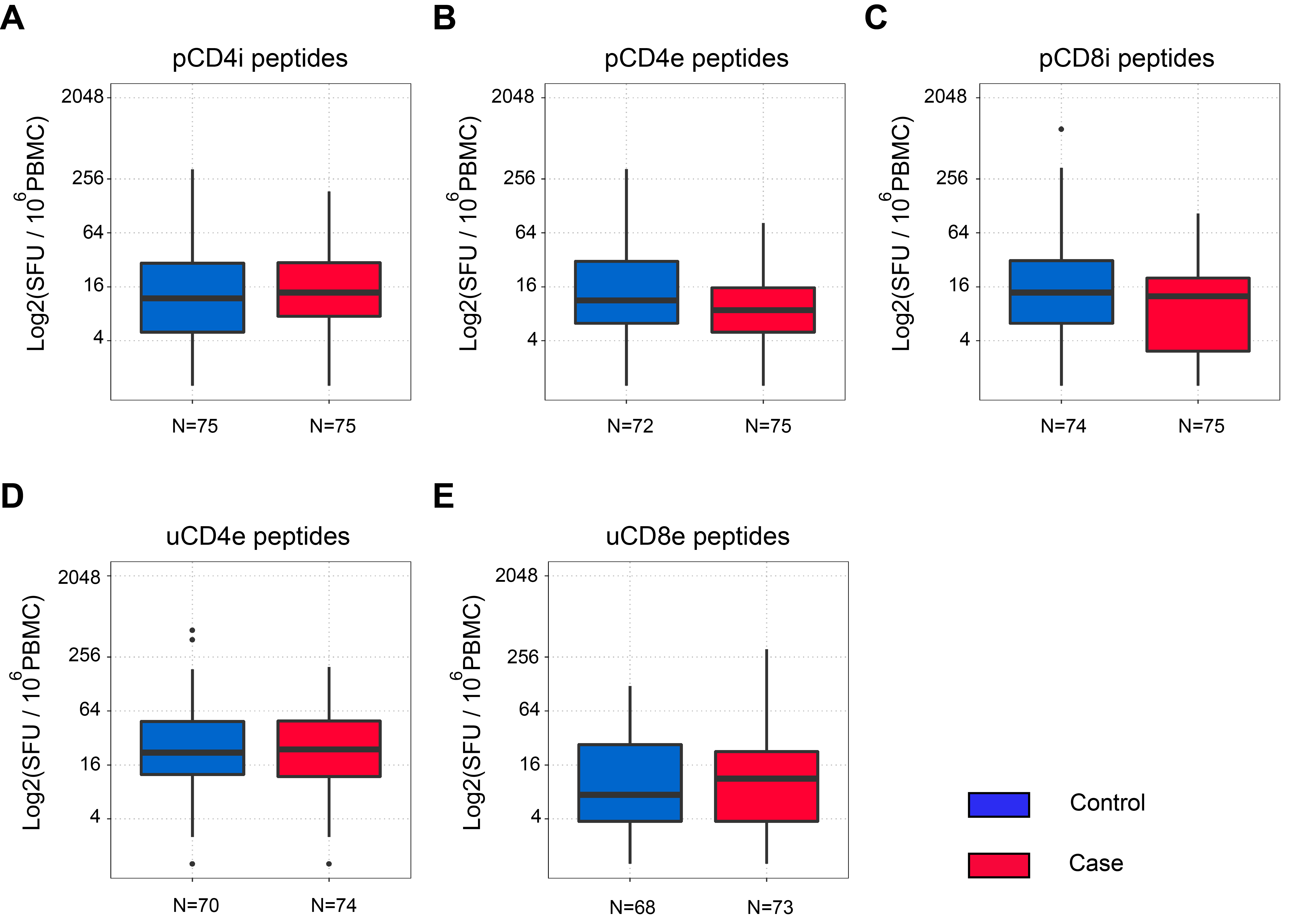

Supplement: S1 Fig — PBMC were stimulated with various influenza A antigen libraries: (A) pCD4i, (B) pCD4e, (C) pCD8i, (D) uCD4e, (E) uCD8e (p values, Wilcoxon-Mann-Whitney test). (TIF) [file pone.0188055.s001.tif]

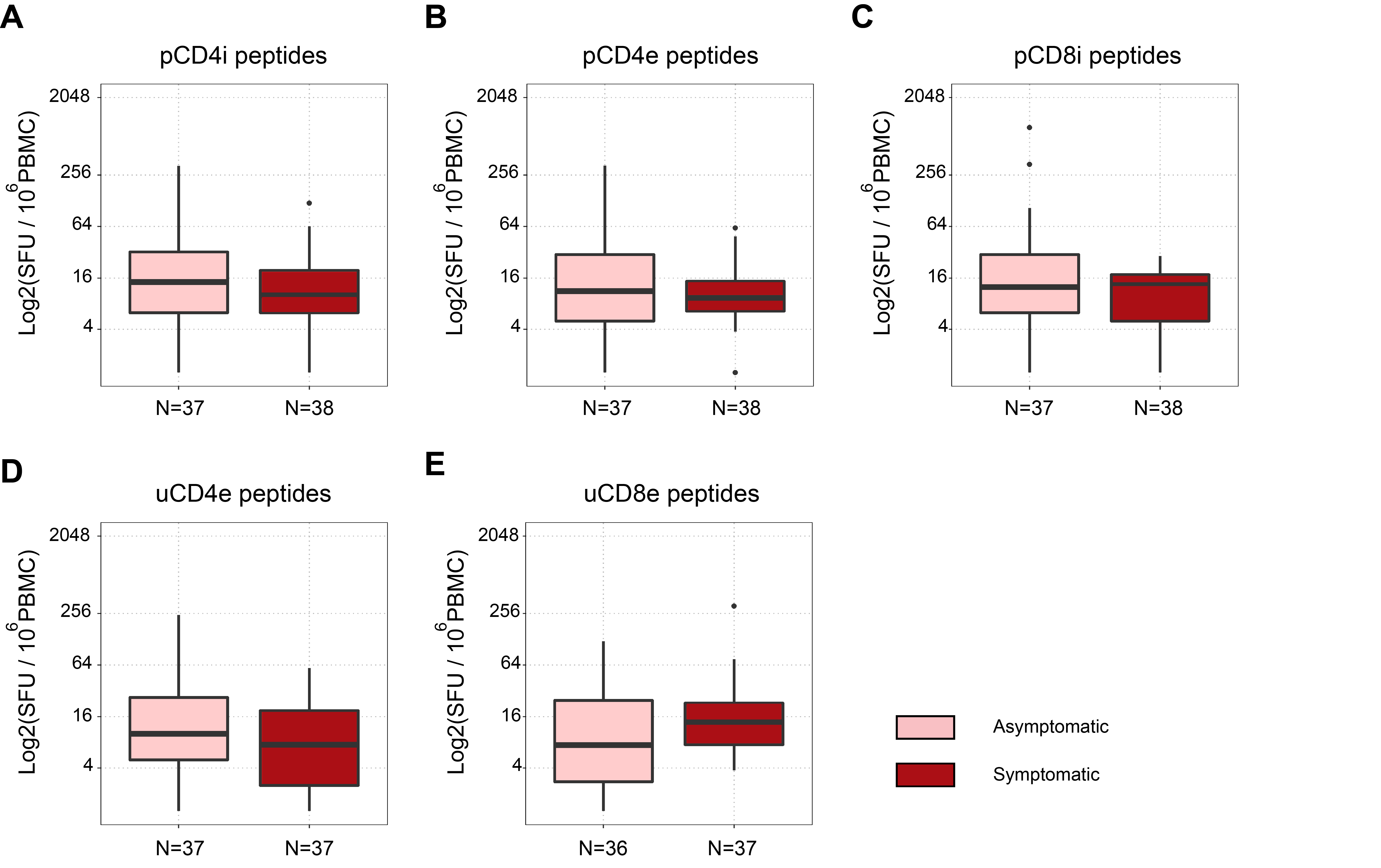

Supplement: S2 Fig — PBMC were stimulated with various influenza A antigen libraries: (A) pCD4i, (B) pCD4e, (C) pCD8i, (D) uCD4e, (E) uCD8e (p values, Wilcoxon-Mann-Whitney test). (TIF) [file pone.0188055.s002.tif]

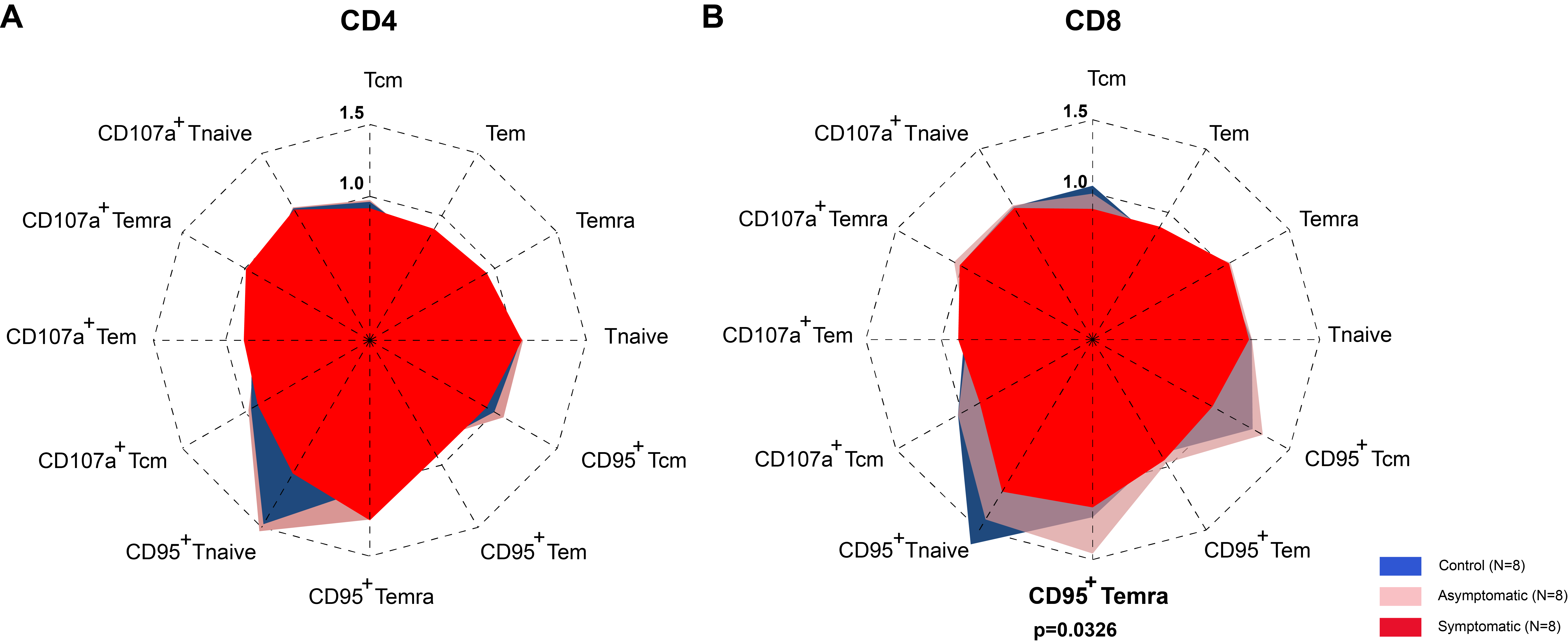

Supplement: S3 Fig — Radar graphs represent median cell frequency fold change of (A) CD4+, (B) CD8+ memory T cells subsets. Dotted lines in graphs represent the fold change level (p values, Kruskal-Wallis test). (TIF) [file pone.0188055.s003.tif]

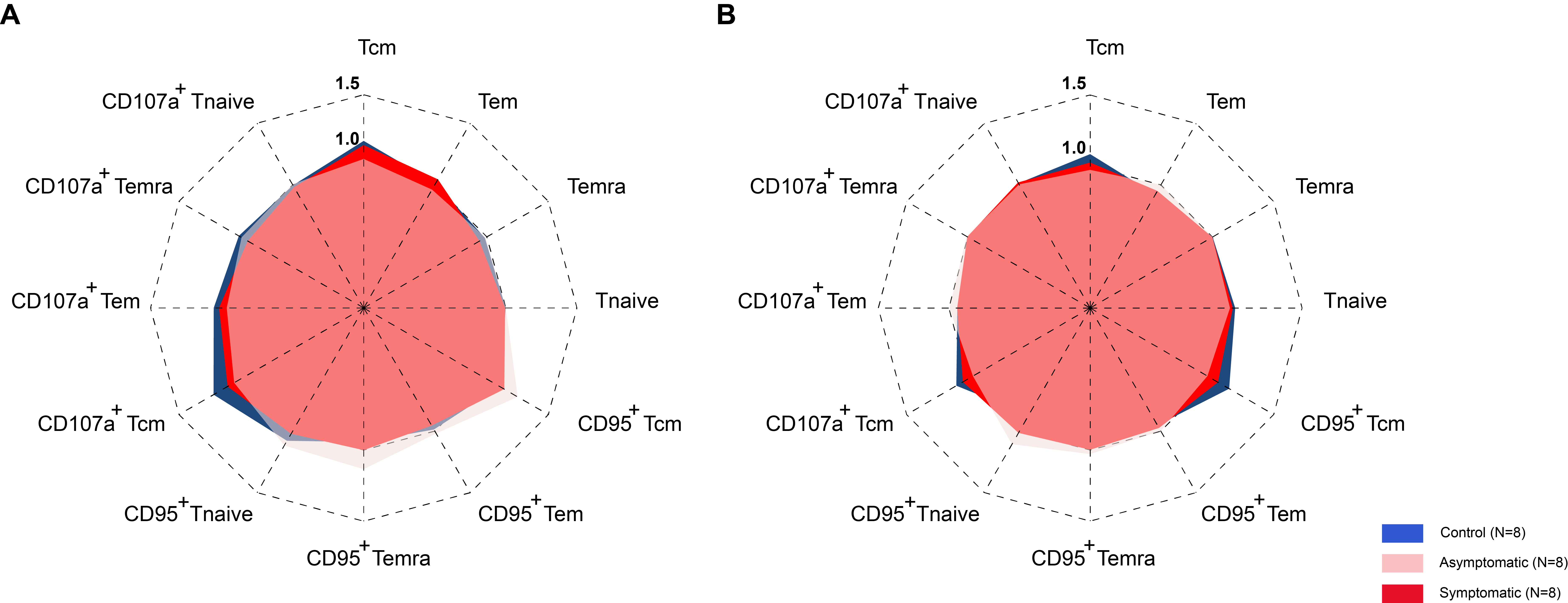

Supplement: S4 Fig — Radar graphs represent median cell frequency fold change after stimulation with (A) uCD8i, (B) CMV epitopes. Dotted lines in graphs represent the fold change level (p values, Kruskal-Wallis test). (TIF) [file pone.0188055.s004.tif]

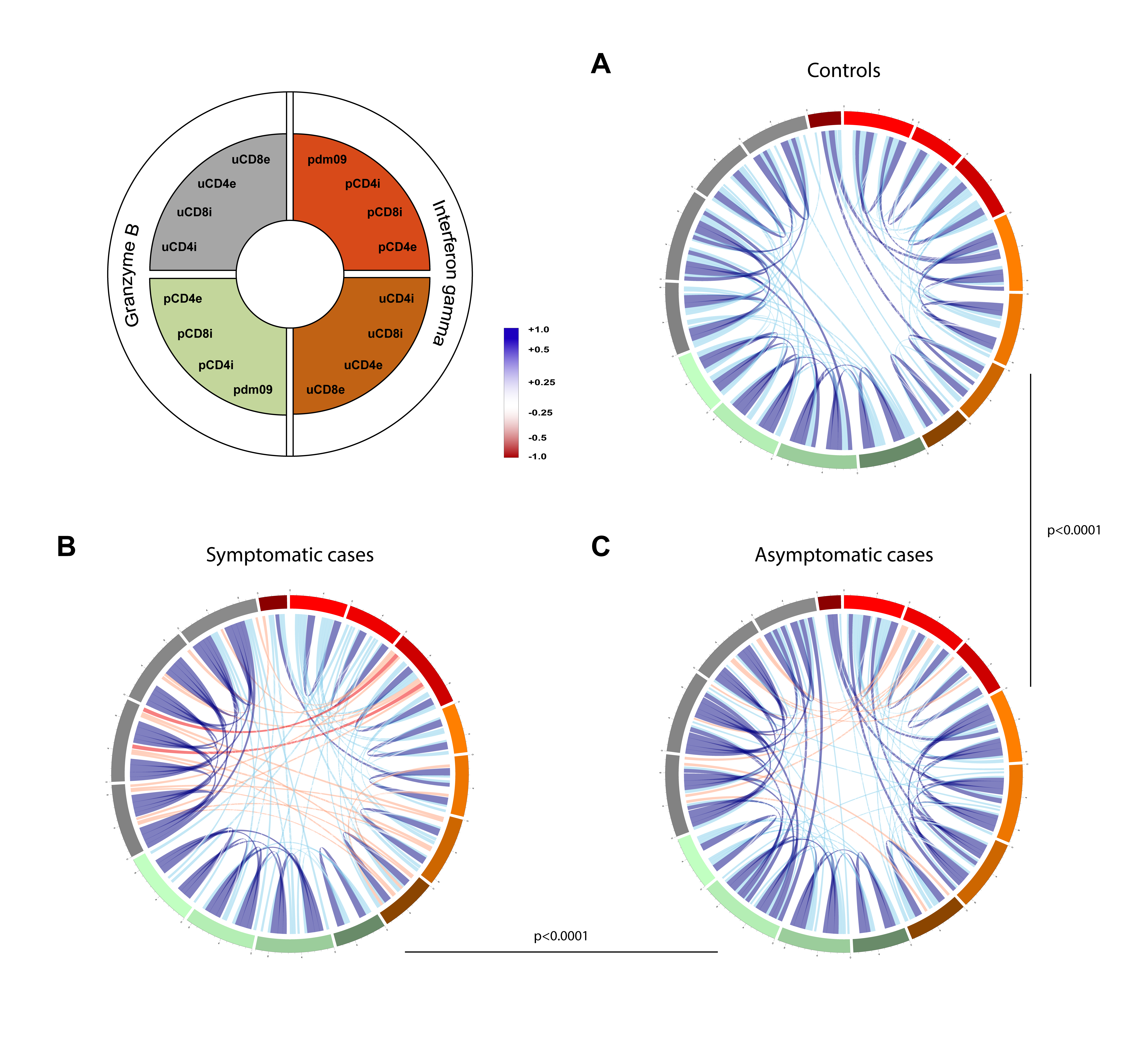

Supplement: S6 Fig — (A) controls, (B) symptomatic cases, (C) asymptomatic cases. Top left diagram represents visual legend for the segments orientation at each diagram–one segment corresponds to a particular PBMC and antigen stimulation combination, whereas left and right semicircles designate Granzyme B and IFNγ PBMC compartments, respectively. For clarity correlation coefficient values between -0.25 and +0.25 were not presented, values between 0.25 and 0.5 in light color, and values between 0.5 and 1 in dark color (see the bar at the top left panel) (p values, Steiger’s test). (TIF) [file pone.0188055.s006.tif]

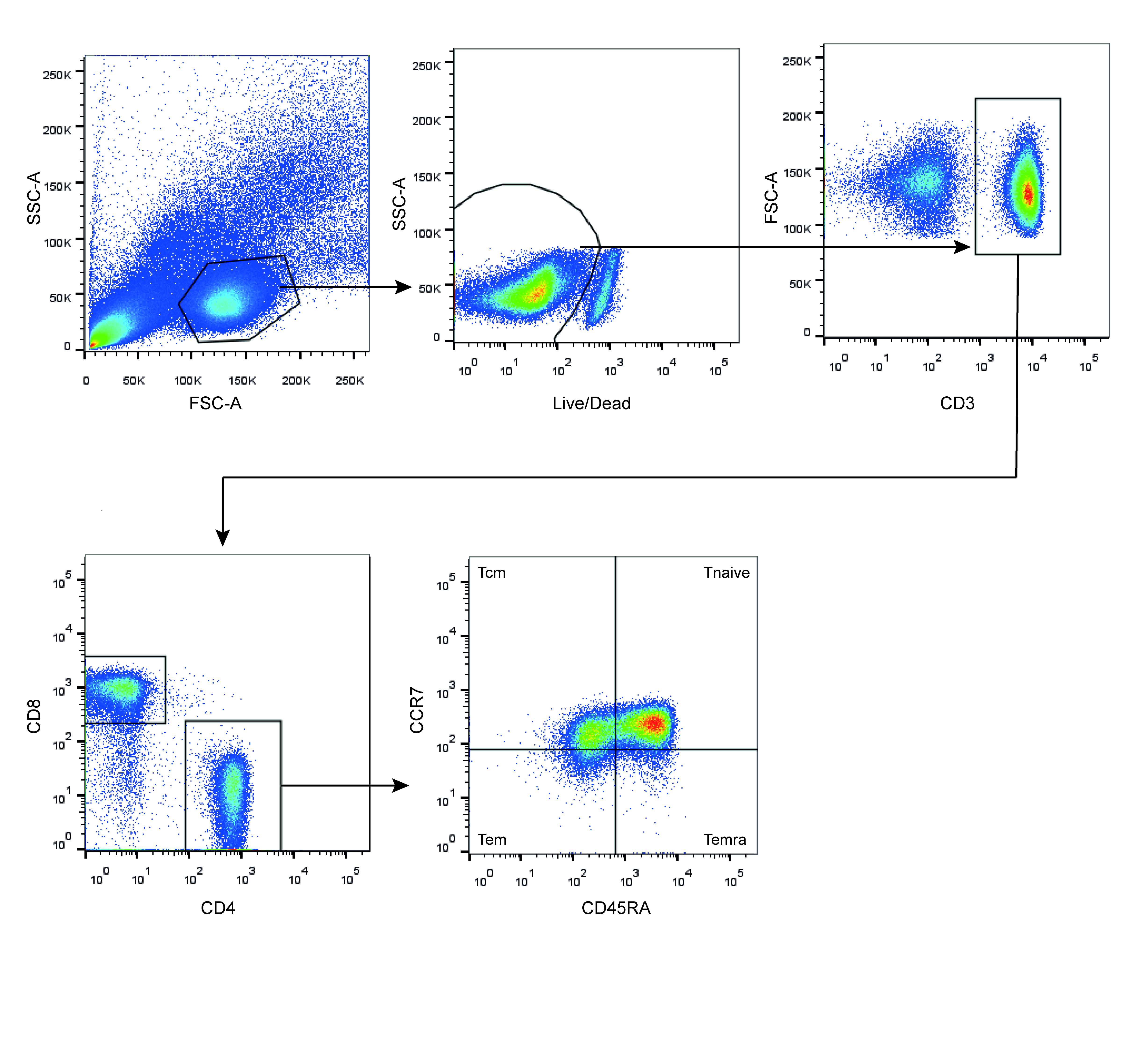

Supplement: S7 Fig — SSC-A–side scatter area, FSC-A–forward scatter area, Live/Dead–aqua live/dead viability dye. (TIF) [file pone.0188055.s007.tif]

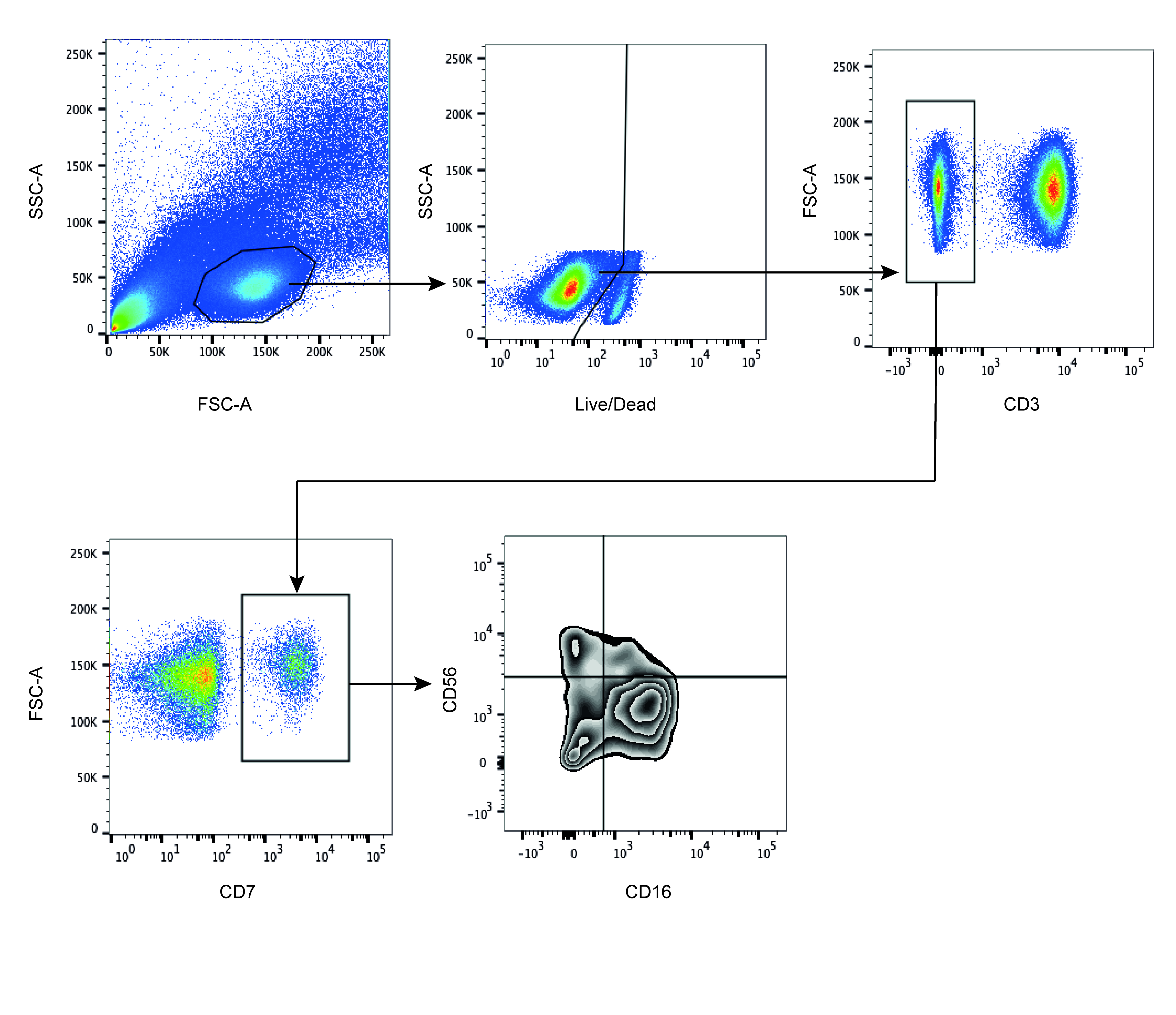

Supplement: S8 Fig — SSC-A–side scatter area, FSC-A–forward scatter area, Live/Dead–aqua live/dead viability dye. (TIF) [file pone.0188055.s008.tif]
