# Supplementary material for: Distinct T and NK cell populations may serve as immune correlates of protection against symptomatic pandemic influenza A(H1N1) virus infection during pregnancy
Source: PLoS One. 2017 Nov 16;12(11):e0188055. doi: 10.1371/journal.pone.0188055 (PMC5690673; doi:10.1371/journal.pone.0188055)
Supplement: S1 Table — (DOCX) [file pone.0188055.s009.docx]

**S1 Table. Demographic and other characteristics of cases and controls.**

| Characteristics of women and children | | Controls | Cases |
| --- | --- | --- | --- |
| Total pregnant women (biological samples used) | | 75 | 75 |
| Median age [years] at delivery (range) | | 32 (17-42) | 31 (19-42) |
| Mean time [months] between the pandemic peak and birth | | 7.5 | 7.4 |
| Marital status | |  |  |
| Married | | 31 | 34 |
| Cohabitating | | 40 | 41 |
| Single | | 2 | 0 |
| Education | |  |  |
| High school graduate or less | | 18 | 11 |
| University education | | 51 | 58 |
| Use of tobacco | |  |  |
| Smoking at the beginning/during pregnancy | | 5 | 1 |
| Father smoking | | 8 | 9 |
| Parity | |  |  |
| 0 | | 42 | 41 |
| 1 | | 20 | 21 |
| >= 2 | | 13 | 13 |
| Health related conditions during pregnancy | |  |  |
| Allergies | Pollen | 14 | 6 |
|  | Animal hair | 5 | 4 |
|  | Other allergies | 4 | 9 |
| Atopic eczema during pregnancy | | 5 | 5 |
| Asthma | | 2 | 1 |
| Diabetes mellitus | | 1 | 2 |
| Arthritis | | 0 | 2 |
| Use of medications during pregnancy | |  |  |
| Antiviral medicines (Tamiflu or Relenza) | | 0 | 5 |
| Antipyretics | | 0 | 6 |
| Antibiotics | | 1 | 2 |
| Children characteristics | |  |  |
| Gender (count) | | Male (46) | Male (44) |
| Median Apgar score at 1min (range) | | 9 (7-10) | 9 (7-10) |
| Median Apgar score at 5min (range) | | 10 (8-10) | 10 (8-10) |
| Mean child weight in g (range) | | 3585 (2480-4700) | 3617 (2693-5200) |
| Mean child length in cm (range) | | 50.8 (45-56) | 50.9 (47-57) |
| C-section delivery | | 14 | 2 |
