# Supplementary material for: Distinct T and NK cell populations may serve as immune correlates of protection against symptomatic pandemic influenza A(H1N1) virus infection during pregnancy
Source: PLoS One. 2017 Nov 16;12(11):e0188055. doi: 10.1371/journal.pone.0188055 (PMC5690673; doi:10.1371/journal.pone.0188055)
Supplement: S2 Table — (DOCX) [file pone.0188055.s010.docx]

**S2 Table. Antibodies used for phenotyping T and NK cells.**

| Memory phenotype^a^ | | Effector phenotype | | NK cells^b^ | |
| --- | --- | --- | --- | --- | --- |
| Marker | Fluorochrome | Marker | Fluorochrome | Marker | Fluorochrome |
| live/dead | AmCyan (aqua) | live/dead | AmCyan (aqua) | live/dead | AmCyan (aqua) |
| CD3 | PercpCy5.5 | CD3 | PercpCy5.5 | CD3 | PercpCy5.5 |
| CD4 | PE | CD4 | PE | CD7 | PE |
| CD8 | APC cy7 | CD8 | APC Cy7 | CCR7 | V450 (PacBlue) |
| CCR7 | v450 (PacBlue) | IL2 | APC | CD16 | FITC |
| CD45RA | FITC | IFNγ | FITC | CD56 | PE7 |
| CD95 | PE7 | TNFα | V450 (PacBlue) | CD107a | APC |
| CD107a | APC | MIP1β | PE Cy7 | IFNγ | PE Cy7 |

^a^ T cell memory subsets were defined as following: late effector (Temra) – CD45RA^+^CCR7^-^, effector memory (Tem) – CD45RA^-^CCR7^-^, central memory (Tcm) – CD45RA^-^CCR7^+^, and naive (Tnaive) – CD45RA^+^CCR7^+^ (see S7 Fig for gating strategy).

^b^ Gating strategy is presented in S8 Fig.
