# Supplementary material for: Distinct T and NK cell populations may serve as immune correlates of protection against symptomatic pandemic influenza A(H1N1) virus infection during pregnancy
Source: PLoS One. 2017 Nov 16;12(11):e0188055. doi: 10.1371/journal.pone.0188055 (PMC5690673; doi:10.1371/journal.pone.0188055)
Supplement: S1 File — Additional details regarding Materials and Methods, and Results section. (DOCX) [file pone.0188055.s011.docx]

Supporting Information

Distinct T and NK cell populations may serve as immune correlates of protection against symptomatic pandemic influenza A(H1N1) virus infection during pregnancy

Miloje Savic, Jennifer L. Dembinski, Ida Laake, Olav Hungnes, Rebecca Cox, Fredrik Oftung, Lill Trogstad, Siri Mjaaland

**Results**

**Study details**

We have included 164 women that were pregnant during the peak of pandemic influenza, among which 82 were exposed to the pandemic virus. In total, 14 samples were of poor quality (low cell counts and/or low viability), and were excluded from further study leaving 75 samples in each case and control group. Demographic characteristics are provided in S1 Table.

**PBMC secretion of IFNγ and Granzyme B after stimulation with various influenza A epitopes**

Controls had higher median frequency of Granzyme B producing cells compared to cases after stimulation with the pdm09 virus (p=0.0257) (results not shown). The median frequency of Granzyme B producing T cells was lower among asymptomatic cases (N=37) compared to those who experienced ILI symptoms (N=38), when PBMC were stimulated with pCD4i epitope library (p=0.0645, results not shown).

Assessing the inter-relationship between IFNγ and Granzyme B expression after stimulation with various influenza A epitope libraries emphasized two general trends (S6 Fig). First, positive correlations were more prominent within each of the two PBMC compartments (defined as PBMC secreting IFNγ or Granzyme B). Second, between two PBMC compartments negative correlations were increasing both in frequency and magnitude when comparing three groups of women (S6 Fig A, B, C). Overall difference between controls and asymptomatic cases showed more negative correlations within and between two PBMC compartments (p<0.0001, S6 Fig A, C), while differences between asymptomatic and symptomatic cases were mostly characterized by negative correlations between two compartments (p<0.0001, S6 Fig B, C). Collectively our results indicate that subtle alternations in the PBMC network are correlated with disease progression and associated symptoms severity.

**S1 Table. Demographic and other characteristics of cases and controls.**

**Materials and methods**

**Study participants**

The cohort holds extensive data from more than 3500 mother and child pairs: self-reported questionnaires and national registry data: exposure to influenza infection (the Surveillance System for Infectious Diseases, MSIS), vaccination (the Norwegian Immunization Register, SYSVAK), and perinatal outcomes (the Medical Birth Registry, MBRN). Self-reported questionnaires from 2010 contain information about general health during pregnancy, information about influenza infection and vaccination, antiviral medication use and influenza illness related symptoms. Around 2600 mother-child pairs have biological samples (plasma, PBMC, RNA and DNA), collected at delivery (between March and October 2010), and deposited at the Norwegian Institute of Public Health’s biobank.

**Ethics approval**

The project protocol was reviewed and approved by the institutional review board at the NIPH and Regional Committee for Medical and Health Research Ethics South East, Norway (study reference numbers 2009/2165 and 2010/2937). All participants have signed a written consent.

**Hemagglutination inhibition assay (HI)**

Assays were performed as previously described [1]. HI antibodies were detected in sera with 0.7% turkey red blood cells and 8 HA units of influenza A/California/07/09(H1N1) virus. HI titers were expressed as the reciprocal of the last serum dilution which induced a complete inhibition of hemagglutination.

***Ex vivo* enzyme-linked IFNγ/ Granzyme B immunospot assay (ELISpot)**

Dual color IFNγ and Granzyme B 96 well plates were used according to the manufacturer’s instructions (CTL, Bonn, Germany). Detailed protocol can be obtained at: dx.doi.org/10.17504/protocols.io.jtncnme. PBMC were isolated using SepMate tubes following manufacturer’s recommendations (Stemcell Technologies SARL, Grenoble, France). Detailed protocol for PBMC isolation and long-term storage can be obtained at: dx.doi.org/10.17504/protocols.io.jtmcnk6. Each well contained 200 000 PBMC in AIM-V medium (Gibco, Thermo Fisher Scientific, Norway). Antigenic stimulants used were: negative control (0.28% DMSO in AIM-V to match the final DMSO concentration in each of the epitope libraries), positive control (Concanavalin A 2μg/ml (ConA)), H2O2 inactivated A(H1N1)pdm09 virus at 75 HAI units [2], and epitope libraries (2µg/ml final concentration per well of individual peptides in DMSO/AIM-V) [3]. Plates were incubated for 20h at 37°C in a humidified incubator with 5% CO_2_ and developed the following day. The plates were read using a CTL S6 Ultra V Immunospot analyzer (CTL, Bonn, Germany), and results presented as spot forming units per one million PBMC (SFU/10^6^ PBMC). All samples were responsive to the ConA positive control.

**Flow cytometry assays**

Frozen PBMC samples were thawed, resuspended in complete media (RPMI-1640, 10% FBS, pen/strep; Gibco, Thermo Fisher Scientific, Norway), and allowed to rest overnight (~18h) in a 37˚C 5% CO_2_ incubator. The following morning cells were stimulated at a concentration of 1x10^6^ per well (96 well plate, Nunc, Thermo Scientific, Norway), for 4 hours in a 37˚C 5% CO_2_ incubator with the following antigens: ConA 5μg/ml, H_2_O_2_ inactivated A(H1N1)pdm09 virus at 75 HAU units [2], uCD8i peptide library (4µg/ml), cytomegalovirus (CMV) control peptides (AnaSpec Inc., Fremont, USA), or corresponding negative control (medium and DMSO). The cells were co-stimulated with anti-CD28 and anti-CD49d monoclonal antibodies at 5μg/ml each (BD Biosciences, Sweden) [4], and 1μl of Brefeldin A (BFA) following manufacturer’s instructions (BD Biosciences, Sweden). Detailed protocol can be obtained at: dx.doi.org/10.17504/protocols.io.jtpcnmn.

Cells were stained using the aqua LIVE/DEAD fixable cell stain (Invitrogen, Life Technologies, Norway), fixed and permeabilized using Perm/Wash (BD Biosciences, Sweden) following manufacturer’s instructions. The list of fluorescently conjugated antibodies (all from BD Biosciences, Sweden) is provided in S2 Table.

**S2 Table. Antibodies used for phenotyping T and NK cells.**

Corresponding compensation controls were prepared using Compbeads (for antibodies; BD Biosciences, Sweden) and ArC amine reactive beads (for LIVE/DEAD stain; Invitrogen), following manufacturer’s instructions.

All samples were responsive to the ConA positive control.

**Statistical analysis**

Missing data were imputed using the mice package in R [5, 6]. In total five sets of missing data were imputed using predictive mean matching method, and variables with more than 25% of missing data were excluded from final analyses. The differences between two correlation matrices were compared using Steiger’s test. Statistical analyses were performed in STATA version 13.0 (STATA Corp. Texas, USA), and R 3.2.2 [7].

**References**

1. Network WGIS, editor Manual for the laboratory diagnosis and virological surveillance of influenza2011.

2. Dembinski JL, Hungnes O, Hauge AG, Kristoffersen AC, Haneberg B, Mjaaland S. Hydrogen peroxide inactivation of influenza virus preserves antigenic structure and immunogenicity. Journal of virological methods. 2014;207:232-7. doi: 10.1016/j.jviromet.2014.07.003. PubMed PMID: 25025814.

3. Savic M, Dembinski JL, Kim Y, Tunheim G, Cox RJ, Oftung F, et al. Epitope specific T-cell responses against influenza A in a healthy population. Immunology. 2016;147(2):165-77. doi: 10.1111/imm.12548. PubMed PMID: 26489873; PubMed Central PMCID: PMCPMC4717245.

4. Horton H, Thomas EP, Stucky JA, Frank I, Moodie Z, Huang Y, et al. Optimization and validation of an 8-color intracellular cytokine staining (ICS) assay to quantify antigen-specific T cells induced by vaccination. J Immunol Methods. 2007;323(1):39-54. doi: 10.1016/j.jim.2007.03.002. PubMed PMID: 17451739; PubMed Central PMCID: PMCPMC2683732.

5. Stef van Buuren KG-O. mice: Multivariate Imputation by Chained Equations in R. Journal of Statistical Software. 2011;45(3):1-67.

6. Zhang Z. Multiple imputation with multivariate imputation by chained equation (MICE) package. Ann Transl Med. 2016;4(2):30. doi: 10.3978/j.issn.2305-5839.2015.12.63. PubMed PMID: 26889483; PubMed Central PMCID: PMCPMC4731595.

7. Team RC. R: A language and environment for statistical computing. R Foundation for Statistical Computing, Vienna, Austria. 2015.
